# Harmonizing Drying Time, Layer Thickness, and Drier Zones for Drying Kinetics: Quality and Safety of Solar Tunnel-Dried Wet-Processed Parchment Coffee (*Coffea arabica* L.)

**DOI:** 10.1155/2023/6677592

**Published:** 2023-09-14

**Authors:** Zenaba Kadir Abdissa, Yetenayet B. Tola, Addisalem Hailu Taye, Hayat Hassen Mohammed

**Affiliations:** Jimma University College of Agriculture and Veterinary Medicine, Department of Postharvest Management, P.O. BOX 307, Ethiopia

## Abstract

Tunnel solar dryer is the recently used drying method for better quality and safety of parchment coffee. However, the higher variation of drying temperature and RH along the long tunnel solar dryer results in a heterogeneous environment in the tunnel, which could make parchment coffee dried at different times or with different moisture contents. This study is aimed at investigating the effect of solar tunnel dryer zones at different zones of the dryer, divided into three zones from the inlet to the exit side of the drier and drying layer thicknesses on the drying time, drying kinetics, physicochemical, sensory, and fungal growth loads of parchment coffee. Furthermore, seven mathematical models were evaluated to select the best-fitting model for a specific zone to predict drying time. Results showed that dryer zones significantly (*p* < 0.05) interacted with layer thickness for most of the measured parameters except titratable acidity and sensory properties. The dryer zone, coupled with the reduction in drying layer thickness, caused an increase in effective diffusivity and moisture removal rate and reduced drying time. The drying time to reach constant moisture content varied from 14 to 17 hours. Overall raw bean, cup, and total quality varied from 36.3 to 37, 48 to 51, and 84.3 to 87.3%, respectively. Values for physicochemical parameters ranged from 5.3 to 6.9 (pH), 2.1 to 2.6% (titratable acidity), 2.3 to 4.3°Brix TSS, 10.9 to 15.2% (ether extract), 39.2 to 53.5GAE/g (total phenolic content), and 38.5 to 59.2 (DPPH scavenging capacity). The fungal infection percentage at the end of drying varied from 4 to 93.3%, which could be associated with potential mycotoxin formation if recommended conditions were not maintained. In general, for better quality, similar drying times, and a lesser fungal load, it is recommended to use 4, 5, and 6 cm layer thickness in zones one, two, and three, respectively. The drying kinetics of parchment coffee in different dryer zones with different drying layer thicknesses showed variation. Zone one at 2 and 4 cm layer thicknesses is best described by the Verma model. Four- and six-centimetre layer thicknesses in zones 2 and 3 are best described by the modified Midilli model.

## 1. Introduction

Globally, coffee is consumed for its refreshing and stimulating effects. Apart from this, studies showed that coffee consumption has several health benefits. The positive effect of habitual coffee consumption with lower risk of certain noncommunicable chronic diseases like type 2 diabetes mellitus, chronic liver diseases, and certain cancer types has been confirmed in many cohort studies in different parts of the world [1]. These types of findings are shifting the general perception of consumers' coffee as a health-promoting beverage rather than a stimulant drink. The stimulating and health benefits of coffee emanate from a complex mixture of chemical and bioactive compounds for the desired simulation effect, aroma, flavour, and other health benefits. Recent meta-analyses demonstrated a beneficial relationship between habitual coffee intake and improved population health [1]. With growing scientific evidence, the perception of coffee has been changed from a luxury stimulant drink to that of health-promoting beverage. An epidemiological study conducted in the UK using large sample size (500,000 participants) showed the presence of an association between the number of cups consumed per day and decreased all-cause mortality [2]. Additional epidemiological studies also indicated a lower risk of several clinical outcomes and of all-cause mortality for habitual coffee consumption [3, 4]. It is indicated that not the caffeine content but rather other phytochemicals in coffee can account for the most beneficial properties of coffee [1]. However, these inherent photochemicals, other health-promoting agents, and other quality characteristics can be affected by different postharvest handling practices unless and otherwise optimum practices are established.

Drying should maintain the color, flavor and taste of the product (Aregba et al., 2006; [5]). However, open sun drying demands longer drying time and does not guarantee protection from the chance of rewetting of the product. The slow drying and rewetting conditions with available free water on the parchment could contribute for possible contamination and growth of mycotoxin-producing fungi ([6]; Musebe et al., 2014) and high risk of mycotoxigenic contamination due to slow drying even after drying [7]. Studies also confirmed that coffee beans are prone to different types of mycotoxin contaminations under different processing methods and environmental conditions [8–10]. Good postharvest practices are recommended to reduce high risk of mycotoxin formation including optimum drying time to desired and safe moisture content. Apart from this, delay in sun drying process adds labour costs to producers. To overcome these limitations, different types of solar driers are often used by large-scale commercial farms. The technology is commonly recommended as an alternative sustainable and affordable method to dry most of the agricultural products [11, 12] with required degree of quality and safety.

Solar drying is one of the green technologies to preserve agricultural products by reducing their moisture content to a level that prevents the growth of microorganisms and other biochemical processes. To develop solar drying technology, the high solar intensity prevalent in tropical regions can be used as a source of solar energy. Among solar driers, solar tunnel drier is one of the most commonly used methods to dry parchment coffee. However, the length of the drier in commercial coffee processing units is often greater than 16 meter. In long drier, it is difficult to get uniform temperature and relative humidity throughout the drier zones. A heterogeneous drying temperature and RH along the drier zones result in variation in drying rate and time for coffee parchments. Parchment coffee in certain part of the long tunnel solar drier may be overdried while parchments in other part still have moisture above the desired level. This will have an impact on the quality and safety of the product at the end of drying time. Variation in drying time along the drier zones makes handling and storage of parchment coffee difficult. However, it is believed that variation in drying rate among different zones can be harmonized by applying different drying layer thicknesses that better fits to specific drier zone and by identifying and applying specific drying kinetic model for better quality and safety. Therefore, this study is aimed at evaluating the effect of different drier zones and drying layer thicknesses on the drying kinetics as a means to solve the limitations of long tunnel solar drier to produce better quality and safe green coffee beans.

## 2. Material and Method

### 2.1. Sample Collection and Preparation

Coffee (*Coffea arabica* L.) fruits were harvested from Saqqa Chokorsa district, Wokito village. The fruits were subjected to wet processing methods, pulped, fermented for 24 hr at room temperature (25 ± 2°C), and finally washed to make the parchment coffee ready for drying experiment. The experiment was conducted at Jimma University College of Agriculture and Veterinary Medicine, Food Science and Postharvest Technology demonstration field, located about 70 33^″^ N latitude and 360 57^″^ E longitudes and altitude of 1710 meter above sea level (m.a.s.l). The mean maximum and minimum temperatures are 26.8°C and 11.4°C, respectively, and the maximum and minimum relative humidities are 91.45% and 39.92%, respectively.

### 2.2. Description of Tunnel Solar Drier and Geographical Location

**Figure 1:**
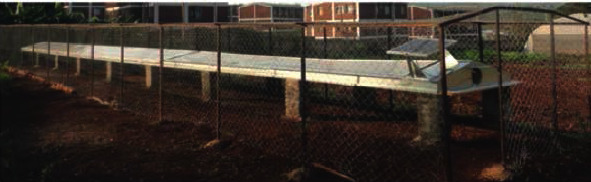
Real picture and schematic diagram showing details of solar tunnel drier used for the study and the three drying zones classified according to temperature and RH variation along the drier from absorber to exit end.

The solar tunnel drier has a length of 24 m and a width of 2 m as indicated in Figure 1. It is laid on red brick stands at a height of 0.8 m. The top transparent part is polyethylene sheet, and the bottom part is a 5 cm thickness insulation layer. The solar absorber and drying zones are 8 and 16 m long, respectively. The fan located at the entrance of the solar drier has a capacity of 75 watts to suck and mix ambient air with air in the absorber section to reduce the very hot temperature created at the absorber before it enters to zone one of the drier. The fan operated using solar panel (WS 80/85 Mono RHA/D, Germany) is attached at the top of the front side of the drier.

Based upon the aim of the research, the drying part is subdivided into three zones, each having 5.33 meters long (Figure 1). The zones were determined after collecting preliminary data on temperature and relative humidity (RH) patterns of the tunnel solar drier with the help of data acquisition devices (Testo, model 184 H1, Germany). The temperature and relative humidity of zone 1, zone 2, and zone 3 of the drier were 45 ± 5°C and 30 ± 5%, 55 ± 5°C and 23 ± 5%, and 65 ± 5°C and 16 ± 5%, respectively (Figure 1). The study was conducted in the College of Agriculture and Veterinary Medicine of Jimma University between December 31, 2020, and January 5, 2021, receiving average direct solar irradiation of 1562.3 kW/m^2^ per year.

### 2.3. Experimental Design and Treatment Combinations

The experiment had two main factors, dryer zones with three levels (zone 1, zone 2, and zone 3) and open sun (as a control), and three levels of drying layer thickness (2, 4, and 6 cm). The experiment was laid as 4 × 3 factorial combination arranged in randomized complete block design (RCBD) and replicated three times in a total having 36 experimental units.

### 2.4. Data Collected

#### 2.4.1. Physical Property and Drying Characteristics

*(1) Temperature and Relative Humidity Measurements*. Ambient air temperature and relative humidity were measured in one-hour interval during the day time (9:00 am to 5:00 pm) inside and outside of the tunnel using a temperature and relative humidity data logger (Testo, 184 H1, Germany).

The moisture content of parchment coffee after washing as initial moisture content and after drying was determined by drying of 5 g randomly picked parchment coffee using the convective oven (Leicester, LE67 SFT England) at 105°C for 16 hours [13]. The average value of MC (% w.b) was determined using

(1) $$\begin{array}{c} \text{MC }\left(\%\right)=\frac{W1-W2}{W1}*100, \end{array}$$

where *W*_1_ is the initial weight (g), *W*_2_ is the final weight after drying (g), and MC is the moisture content (%).

*(2) Moisture Ratio*. Concerning drying data analysis, the moisture ratio (MR) is essential for describing the various models of thin-layer drying and determined using [14]

(2) $$\begin{array}{c} \text{MR}=\frac{\left(M-M_{\text{e}}\right)}{\left(M_{0}-M_{\text{e}}\right)}, \end{array}$$

where MR is the dimensionless moisture ratio, *M* is the moisture content at any time (%), *M*_e_ is the equilibrium moisture content (%), and *M*_0_ is the initial moisture content (%) wet basis.

However, MR is simplified to *M*/*M*_0_ in place of the above equation since the relative humidity of the drying air fluctuated continuously under the sun drying [15]. The value of *M*_e_ is relatively small for long drying times, compared to the values of *M* and *M*_0_, so the equation can be simplified to *M*/*M*_0_ [16].

#### 2.4.2. Drying Kinetics

*(1) Drying Kinetic Models*. Seven thin-layer drying models (Table 1) were selected to describe the drying behaviour of parchment coffee beans. These models were selected due to their less complicity and commonly used to describe drying behaviour of most agricultural products. The best-fitting model was selected based on its *R*^2^ value, root mean squared error (RMSE) (Eq. (3)), and chi-squared (*χ*^2^) (Eq. (4)); the *R*^2^ value was computed using the RSQ function of Microsoft Excel. For the best fit, *R*^2^ value should be higher and RMSE and *χ*^2^ values should be lower [14].

(3) $$\begin{array}{c} \text{RMSE}=\sqrt{\overset{N}{\underset{t=1}{\sum }}\frac{{\left(\text{M}{\text{R}}_{\text{pre},i}-\text{M}{\text{R}}_{\exp,i}\right)}^{2}}{N}}, \end{array}$$

(4) $$\begin{array}{c} x^{2}=\sqrt{\overset{N}{\underset{t=1}{\sum }}\frac{{\left(\text{M}{\text{R}}_{\exp,i}-\text{M}{\text{R}}_{\text{pre},i}\right)}^{2}}{N-n}}. \end{array}$$

*(2) Effective Diffusivity*. The effective moisture diffusivity was obtained from the plot of ln MR versus drying time. The slope of the graph of natural logarithm experimental moisture ratio versus drying time given is as −Deff/4*L*^2^. Knowing the experimental moisture ratio and the parchment coffee thickness, the effective moisture diffusivity was calculated.

### 2.5. Chemical Properties of Dried Parchment Coffee Beans

#### 2.5.1. pH and Total Soluble Solid (TSS) Content

pH value was determined according to [24]. Total soluble solids of the powdered sample were determined according to [25].

#### 2.5.2. Crude Fat Content

The ether extract content of the dry samples was performed gravimetrically following the AOAC procedure of Soxhlet's method (960.39) [26].

#### 2.5.3. Total Phenolic Content (TPC)

Total phenolic content of green coffee bean powder was determined by using the Folin-Ciocalteu reagent according to the colorimetric method [27]. A calibration curve was made from gallic acid standard solution (100, 150, 200, 250, and 300 mg/L) (*R*^2^ = 0.989) and the blank prepared with distilled water. The total phenolic content was expressed as milligram gallic acid equivalent (GAEs) per dry weight material using the standard curve.

#### 2.5.4. DPPH Scavenging Capacity

The free radical scavenging capacity (RSC) of the extracts was evaluated by DPPH radical assay, as described by [28]. The percentage of radical scavenging activity (RSC) of the tested extracts was calculated using

(5) $$\begin{array}{c} \text{RSC }\left(\%\right)=\frac{\left(A\text{ control}-A\text{ sample}\right)}{A\text{ control}}*100, \end{array}$$

where *A* control and *A* sample are the absorbance values of the control and the test sample, respectively.

Moreover, to compare the radical scavenging efficiency of the extracts, the IC_50_ value indicating the concentration that caused 50% scavenging of the DPPH radical was calculated from the graph plotted by radical scavenging capacity percentage against the extract concentration.

### 2.6. Raw Bean and Cup Quality

#### 2.6.1. Raw Bean Quality

First, the moisture content in the samples was measured on the same day of analysis using the digital electronic moisture taster (mini CAG, Germany). Based on the given parameter, raw quality was assessed for shape and makes color and odor. The raw quality was assessed from primary and secondary defects (10% each), color (10%) and shape (10%). The total raw bean quality score was reported out of 40% [29, 30].

#### 2.6.2. Cup Quality

Cup quality was performed according to the Ethiopian Commodity Exchange Protocol for washed coffee. Freshly boiled water was poured onto the ground coffee up to about half the size of the cup, followed by stirring the content to ensure the homogeneity of the mixture before filling the cup to full size; the volatile aromatic quality and intensity parameters were evaluated by sniffing by five trained panellists. Then, cups were filled to the full size (180 mL) and left to settle, the floater was skimmed, and the brew was ready for cup tasting. Liquor was evaluated for acidity, body and flavour, and mean score values of each variable by the panel used for statistical analysis. Five cups per sample were prepared for each tasting session, and the test was replicated three times and arranged at random. The sensory evaluation of each sample and the cup quality was carried out by a group of five trained panellists and evaluated out of 60%, which is the sum of cup cleanness, acidity, body, and flavour, each of them contributing 15% of the overall scores.

### 2.7. Fungal Infection Percentage

Fungus isolation was performed by direct plating technique on PDA medium (potato extract 4 g/L, dextrose 20 g/L, agar 15 g/L, and distilled water 1 L) according to [31]. Determination of fungus genus was conducted by studying the morphological analysis macroscopically by visualization and microscopically by observing a piece of mycelium and conidial head using the binocular microscope (objective ×40). The infection percentage (%) of coffee beans was determined as the ratio of infected seeds over the total number of coffee seeds tested, as indicated in

(6) $$\begin{array}{c} \text{Mean ratio of seed infection}=\frac{\text{no}.\text{of seed on which fungal species identified}}{\text{no}.\text{of seed tested}}*100. \end{array}$$

### 2.8. Data Analysis

Data were subjected to the analysis of variance (ANOVA) using Minitab version 16 statistical computer software program. Tukey's test at 95% confidence level was used for treatment means separation whenever significant difference occurred. For the determination of drying characteristics, Microsoft Excel Solver add-ins were used to determine the constant values.

## 3. Results and Discussion

### 3.1. Drying Temperature and Relative Humidity during Drying Time

**Figure 2:**
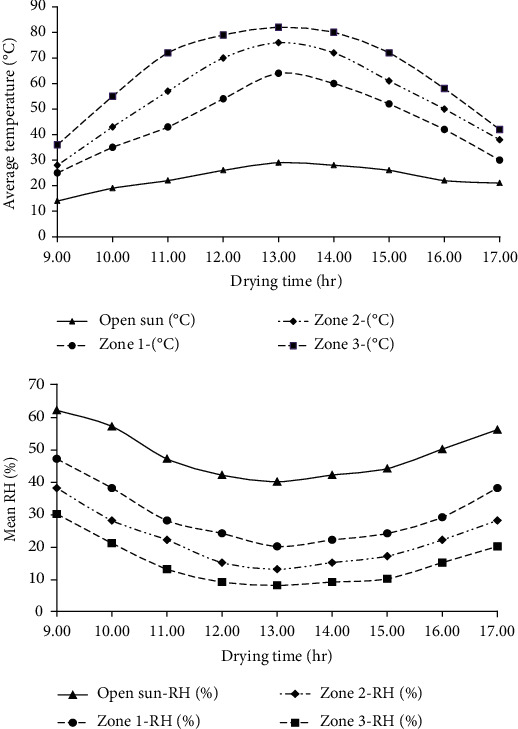
Average hourly temperature (°C) and relative humidity (RH) (%) of drying zones and pen sun drying starting from 9:00 am to 17:00 pm.

Figure 2 shows the recorded average ambient and dryer temperatures (symbols) and relative humidity (solid line with symbols) during the drying periods starting from 9:00 am to 17:00 pm. The average temperatures and RH (%) recorded during the drying period from 09:00 am to 17:00 (between December 31, 2020, and January 5, 2021) were 23 (47%), 45 (30%), 55 (23%), and 64°C (15%) for ambient, zone one, zone two, and zone three, respectively. The result shows that the average temperature in the drier increased by about 10°C along the solar drier from zone one to zone three. Meanwhile, the RH decreased from 7 to 9% corresponding to the temperature. Tesfaye and Habtu [32] conducted the design and performance evaluation of eight-meter tunnel solar drier. They documented the temperature and relative humidity variation over time along the solar dryer. They observed that at the collector end, temperature of the drying medium varied from 33 to 77.9°C, on average of 55.5°C, but the RH varied from 26.2 to 36.3% which is in close agreement with this study. The variation might be associated with the length of tunnel solar drier used for their study. In addition to this, our result is also in agreement with the finding of Ehtenesh et al. (2022) which investigated the effects of solar tunnel drying zones and slice thickness on the drying characteristics of taro (Colocasia esculenta (L.) Schott) slices. Therefore, as compared to open sun drying, the solar tunnel dryer, therefore, generates relatively higher air temperatures and lowers relative humidity to improve drying rates of parchment coffee to shorten the drying time regardless of the zones.

### 3.2. Effect of Dryer Zones and Layer Thickness on Drying Characteristics

#### 3.2.1. Hours of Drying

Table 2 shows significant (*p* < 0.05) variation among drying zones and level of layer thicknesses. Coffee parchment dried in zone 3 with drying layer thickness of 2 cm relatively took the shortest drying time (14 hr), as compared to the longest drying time (47 hr) for coffee sample dried in open sun at a layer thickness of 6 cm. The result is in line with other work [33], which indicates that the longer drying time might not be good for coffee quality and safety point of view. The longer drying time could provide sufficient time growth of mycotoxin-producing fungi due to sufficient moisture and optimum temperature conditions. However, in solar drier, regardless of drier zones, the increase in air temperature and lower RH resulted in a greater water vapour difference, which makes moisture removal easier and quicker [34] as compared to open sun drying.

When drier zones are considered, they exhibited significant difference (*p* < 0.05) in drying time for different layer thicknesses. The higher drying medium and lower RH in zone three for 2 cm layer thickness provided the shortest drying time (14 hr) to reach optimum moisture content. For the same layer thickness, zone two, zone one, and open sun drying however demanded 20.7, 25.3, and 32.7 hours to attain the required moisture reduction for safe storage.

The last column of Table 2 shows the drying time reduction (%) along the drier zone as compared with layer thickness of open sun drying. For instance at 4 cm layer thickness, approximately 23, 43, and 53% reduction in drying time could be achieved as compared to open sun drying at zones one, two, and three, respectively. However, variation of drying temperature and relative humidity at different zones on drying time can be compensated by varying the drying layer thicknesses to achieve similar drying time and moisture content than applying the same layer thickness along the drier. For instance, the layer thickness of 4, 5 (interpolated), and 6 cm can result in an equal drying time of 28 hr to target moisture content in zones 1, 2, and 3, respectively. Adjusting different coffee parchment layers at different zones of the drier results in uniformly dried coffee beans for better quality.

#### 3.2.2. Drying Kinetics of the Parchment Coffee in the Three Zones

**Figure 3:**
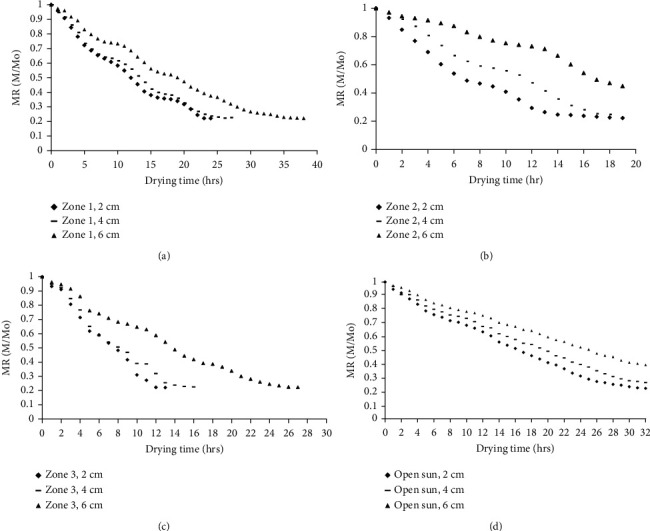
Drying behaviour of coffee parchment due to variation of different drying layer thicknesses in the same drier zone and for parchment coffee dried in open sun as control (a–d).

**Figure 4:**
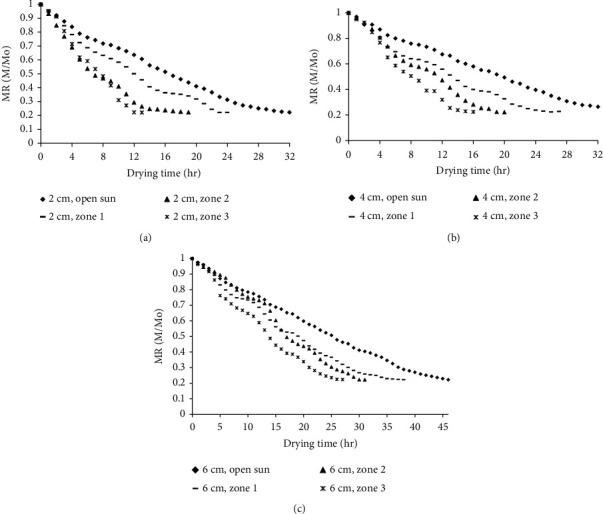
Drying behaviour of coffee parchment due to variation of different drier zones at constant drying layer thickness of 2 (a), 4 (b), and 6 cm (c).

*(1) Effect of Dryer Zones on Drying Behaviour at Constant Layer Thickness*. Dryer zones show variation in terms of rate of drying of parchment coffee. The variations were indicated in Figures 3(a)–3(c) for constant layer thickness. For all layer thickness, the fastest rate was observed in zone three followed by zones two and one (Figures 3(a)–3(c)). The mix-up of relatively cooler ambient air temperature with heated air by the absorber at the first zone could contribute for the relative lower drying air temperature and higher RH as indicated in Figure 2. However, as the length of drier increases, the accumulation of heat with time increases and results in an increase in drying air temperature with reduced RH for faster drying rate. As indicated in Figures 4(a)–4(c), the rate of drying increased from zone three > zone two > zone one. For instance, to reach a moisture ration of 0.3 at layer thickness of 4 cm, there was a need of 12, 15, and 21 hours for zones three, two, and one, respectively (Figures 4(a)–4(c)). This implies the duration of drying to reach the desired moisture content at different zones of the drier not equal and could lead to an implication on quality of the coffee due to over- or underdrying. An increase in vapour pressure difference along the drier length (from zone 1 to zone 3) could contribute for the rapid removal of moisture at constant layer thickness. The accelerated moisture removal could be associated with enhanced kinetic energy of the water molecules for faster surface diffusion at relatively higher drying air temperature and RH as indicated in Figure 2 [35].

The rate variation along the drier could affect the quality of coffee from both over- and underdrying point of views unless and otherwise managed by adjusting drying layer thickness. Underdried coffee beans (>12% w.b), for instance, in zone one, could result in high moisture content which could affect the storage stability as well as quality of the product. Underdrying could result in quality and safety deterioration during storage due to potential enzymatic process and growth of bacteria, mould, and yeast. The same is true in the case of overdried beans (<9% w.b), for instance, in case of zone three, which could have a negative effect to the coffee quality. A one percent reduction in moisture content has also a price implication by the same rate during marketing. From the quality point of view, overdrying could also affect cup quality in terms of aroma, acidity, flavour, and taste losses. In addition to this, the raw bean becomes too brittle (break easily when milled), and its freshness and color can be faded. Overdried coffee bean could be over roasted due to its less moisture content which can be translated in terms of quality and weight (economic) losses. Sandeep et al. [36] reported that, for better quality, the drying temperature for Robusta parchment coffee should not be dried at a temperature greater than 40°C. This result is in close agreement of zone two of the drier despite variation in terms of coffee type studied.

*(2) Effect of Layer Thickness on Drying Behaviour at Constant Dryer Zones*. During the drying of coffee beans at three drier zones with open sun condition, there was a reduction of moisture content from 108.3 to 13% (d.b) for different layer thicknesses. The drying curve of each treatment at a constant dryer zone with different layer thicknesses is indicated in Figures 3(a)–3(d).

The moisture ratio curve for all thickness under all drier zone and open sun condition showed a similar pattern; thus, as the drying thickness increased, the moisture removal rate decreased. In all zones, drying is characterized by a higher rate of moisture removal at the initial drying period, but as drying proceeds, there was a gradual reduction in the rate of moisture removal. As expected, the longer moisture ratio curve was observed for 6 cm layer thickness under all drier zones. This might be due to limited heat and mass transfer rate from the thicker layer which could limit the migration of moisture from the inner layer to the surface. Results from this work are also in agreement with the work of Menya and Komakech [37]. They indicated the higher moisture removal rate for the coffee dried at the lower loading density.

#### 3.2.3. Evaluation and Selection of Suitable Drying Kinetic Models

As indicated in Table 3, a model described by lower RMSE and chi-square as well as higher *R*^2^ values best describes the drying kinetics of parchment coffee at the different drier zones and layer thicknesses. Drying kinetics of parchment coffee at zone one for layer thickness of 2 and 4 cm Verma model [23] described best as compared to the others. However, in the same zone for layer thickness of 6 cm, modified Midilli model [17] fitted well. Two terms model [22] described well the kinetics at zone 2 for a layer thickness of 2 cm. However, for the remaining layer thickness in zone two and for all thicknesses in zone 3, modified Midilli model [17] fitted well to describe the kinetics (Table 3). Therefore, in most of the cases, this model best described the drying characteristics of the parchment coffee in tunnel solar drier. The present study is in agreement with the work of [38] who reported that modified Midilli model is a powerful model in predicting the drying behaviour of parchment coffee within the drying air temperature range of 50-70°C. The drying kinetics of 2 cm layer thickness under open sun condition described well also by the same model, but the diffusion model [18] fitted well for the remaining layer thicknesses for the same drying method.

#### 3.2.4. Effective Moisture Diffusivity

Table 4 shows the values of effective diffusivity for different zones and layer thicknesses. The effective diffusivity varied between 1.2 and 5.0 × 10^−6^ m^2^/s. The highest value was observed for zone three, followed by two, one, and open sun, respectively, as expected from the above discussed data. It is directly proportional to the level of drying medium temperature and RH which were the highest and lowest at zone three. Results in this work are also in close agreement with the work of [39]. They indicated that effective diffusivity decreased with an increase in drying density. They studied the drying characteristics of parchment coffee under solar tunnel drier at different temperatures (30, 40, and 50°C) with an effective diffusivity coefficients between 1.88 × 10^−6^ and 2.26 × 10^−6^ m^2^/s.

### 3.3. Effect on Physicochemical Properties

#### 3.3.1. pH

Treatment combinations showed significant (*p* < 0.05) differences of pH values of parchment coffee. The values varied from 5.3 for sun dried at 6 cm layer thickness as compared to the highest value of 5.9 for coffee dried at 2 cm layer thickness in zone two of the drier. This implies that parchment coffee bean pH can be affected by drier zones in the tunnel solar drier as well with layer thickness during drying. The lowest value might be associated with delay in drying time in open sun drying at 6 cm layer thickness which might contribute for mild fermentation of the beans. Similar result is also reported in Silva et al.'s [40] work, which indicated that the reduction in pH is associated with an increase in fermentation time. The acids produced during fermentation such as acetic acid may penetrate the husks of the coffee bean influencing the changes observed for pH. The rest of the treatment combinations recorded pH value between 5.3 and 5.6 even though they are statistically different. The present finding is in close agreement with the finding reported with [41, 42] who indicate a pH range of 5.3 to 6.52 and 4.89 to 5.98 for green coffee beans, respectively. The pH values indicated in this study are more or less initial pH of parchment coffee beans before fermentation steps. These days' parchment coffee beans are subjected to drying directly from pulping and demucilaging steps by passing the common fermentation process of wet processing. Decrease in the pH of parchment coffee during fermentation time for Arabica (5.43 to 4.71) and Robusta coffee (5.54 to 4.05) was reported in [43]. This implies that fermentation process contributed significantly for pH reduction as compared to drying zones and layer thicknesses observed in this study. Even though significant variations were observed on pH value, for all treatment combinations in this study, the pH values were above 5.2 units.

#### 3.3.2. Total Soluble Solid

The interaction effects of drying zones and layer thickness had a significant (*p* < 0.05) effect on the total soluble solid content of the coffee (Table 5). Thus, the coffee dried under zone two with a layer thickness of 4 cm resulted in the highest (4.3°Brix) score, followed by the coffee dried in zones two and three with a layer thickness of 2 cm (2.9°Brix) each. The lowest total soluble solid content (2.3°Brix) was recorded from the coffee dried under the open sun with a layer thickness of 6 cm. This might happen because coffee dried at a layer thickness of 2 cm was the fastest to dry both under solar tunnel drier and open sun condition. This is because it takes less time to remove the necessary level of moisture content for samples of low layer thickness, which results in reduced TSS loss. The present result is in agreement with the works of [44] who reported that there is an increase in total sugar and soluble sugar contents during drying of coffee at a temperature of 50-60°C which is comparable to drying temperature in solar drier zone 2.

#### 3.3.3. Crude Fat Content

As compared to drying layer thickness, drier zones showed a significant effect (*p* < 0.05) on crude fat content of parchment coffee beans. Result in Table 5 shows that the highest crude fat content was for coffee dried under open sun drying method. However, layer thicknesses showed no significant (*p* > 0.05) effect under the same method. In general, the crude fat content decreased in open sun (14.7-15.1% d.b) > zone 1 (13.5-14.1% d.b) > zone 2 (13.7-13.9% d.b) >zone 3 (10.9-13.2% d.b) with the lesser effect of layer thicknesses. The result in this work is also in agreement with earlier works indicating that the crude fat content of green coffee beans is in the range of 9-16% [45, 46]. However, the decrease in crude fat content along the drier zones might be associated with an increase in drying temperature from open sun to zone 3 (Figure 2) which could result in degradation of heat sensitive fatty acid components. Particularly, an increase in drying temperature with smallest layer thickness of 2 cm in zone 3 results in the lowest value. Since the fat content of coffee is one of desirable quality characteristics, it is necessary to optimize layer thickness along drier zones to minimize the extent of crude fat degradation.

#### 3.3.4. Total Polyphenol Content

Polyphenols are secondary metabolites that plants produce to protect themselves from other pathogenic microorganisms. They play great roles in human health by protecting our body against several diseases related to oxidative stresses and free radical-induced damages [47]. Results of this study show that both drier zones and layer thickness had a significant effect (*p* < 0.05) on polyphenol content, which varied from 39.2 to 53.5 mgGAE/g (Table 5). Results from this work are also in close agreement with the works of [48].

As indicated in Table 5, regardless of drier zones, layer thickness of 4 cm showed remarkable results in total polyphenol content. However, values in this study showed a decrease in total polyphenol content along the drier zones for layer thicknesses of 2 and 6 cm. It was noticed that 4 cm layer thickness retained total polyphenol contents of zone two (53.5)> zone three (50.6)> zone one (49.4)> open sun (48.0 mgGAE/g). The same trend was followed by layer thicknesses of 2 and 6 cm. This implies that both lowest (2 cm) and highest (6 cm) thicknesses result in a negative impact on total polyphenol content either due to impact of drying temperature or fermentation of beans during drying.

#### 3.3.5. DPPH Scavenging Capacity

The interaction effect of drier zones and layer of drying thickness had a significant (*p* < 0.05) effect on DPPH scavenging capacity (Table 5). When observed, in all zones, including open sun drying, 4 cm layer thickness results in relatively better radical scavenging capacity as compared with the other two layer thicknesses. This shows that regardless of drying zones and open sun drying, both fast and slow drying processes associated with 2 and 6 cm layer thicknesses could contribute to the loss of antioxidant compounds due to the impact of drying temperature for 2 cm thickness or fermentation for 6 cm thickness. The values in the table that correspond to the high total phenolic content tend to present a high level of antioxidant activity. Yashin et al. [49] indicated that phenolic compounds in coffee bean are major contributors to their antioxidant capacity. The present work is in agreement with the works of [50] who indicated that Arabica coffee beans consist of coffee oils that are sensitive to heat and moisture which leads to degradation and decrease in the antioxidant activity.

#### 3.3.6. IC_50_ Values

The IC_50_ value for the DPPH scavenging assay was calculated for the treatment combinations. Inhibition concentration value, defined as the concentration of antioxidant required for 50% scavenging of DPPH radicals, is a parameter used to measure antioxidant activity. The smaller IC_50_ value corresponds to a higher antioxidant activity of the plant extract [51]. In the present study, lower IC_50_ value (0.8 mg/mL) was observed in treatment zone two with a layer thickness of 4 cm which indicated its powerful free radical scavenging ability. The highest value of IC50 (6.3 mg/mL) was recorded for the sample layer thickness of 6 cm dried in zone three. IC_50_ showed a similar trend with total phenol content as the highest total polyphenol content related to the highest antioxidant and thus lowest IC_50_ value.

### 3.4. Effects on Sensory Property

The cup quality of coffee is a highly complex trait and depends on physical and sensory qualities, with the coffee variety, climatic conditions during the plant growth, processing method, methods and extent of drying, and storage conditions [52]. The two-way interactions among drier zones and levels of layer thickness showed no significant difference for the total raw quality of coffee (Table 6). All the coffee dried under different drying zones and layer thickness showed statistically similar results.

The total cup quality (Table 6) also showed no significant difference (*p* > 0.05) for the interaction effect of drying zones and the level of drying thicknesses. Even though there is no significant difference between the treatments, coffee dried under zone two with drying thickness of 2 and 4 cm resulted in better score (51.0) among all. From this study, it can be seen that drying in solar tunnel drier resulted in relatively better cup score, when compared to drying under open sun even though they are not significantly different. This could be due to the fact that washed coffees are of high quality resulting in good cup quality and grade in current evaluation criteria.

The total quality of a coffee is the overall quality of the coffee based on the overall quality attribute results used to determine and evaluate the quality potential of coffee. Although statistically similar, the highest overall coffee quality is recorded for coffee parchment dried under zone 2 with drying layer thickness of 4 and 2 cm (87.3) as per the evaluation of panellists. This finding is in line with the result reported by [53], who suggested that the wet processing method resulted in high mean values for good coffee quality. Similarly, [54] showed that solar drying could be an economical and effective method in producing high-quality coffee if coffee drying under good conditions to desired moisture content.

### 3.5. Influence of Drier Zones and Layer Thicknesses on Fungal Growth

Coffee cherries and beans are subjected to contamination and, consequently, colonization by microorganisms during different phases of processing from harvesting to storage. The contamination of coffee beans by fungi affects both the quality in terms of flavour and aroma of the beverage and presents a safety risk to the final product due to the production of toxic secondary metabolites, the mycotoxins, which can be harmful to consumers at certain concentrations [40, 55].

Regarding fungus growth associated with the parchment coffee, a total of 252 fungi were isolated and grouped under *Fusarium*, *Aspergillus*, and *Penicillium* genera. *Aspergillus* was the dominant (186, 73.81%) followed by *Penicillium* (41, 16.27%) and *Fusarium* (25, 9.92%). Similarly, different authors detected these fungi from coffee seeds with parchment [6, 56].

Considering effects of different treatment combinations on the incidence of the microbes, a significant difference (*p* < 0.005) was observed between the treatment combinations. Accordingly, the highest mean incidence (93.3%) was observed on parchment coffee dried under the open sun with a layer thickness of 6 cm. But the lower mean incidence (4.0%) was on parchment coffee dried under zone three with a layer thickness of 2 cm (Table 7).

Layer of drying has shown to have a significant influence on the percentage fungal incidence of the parchment coffee. It is observed that as layer thickness increased, the incidence also increased proportionally under all drying conditions. On the other hand, the fungal incidence decreased with an increase in drying temperature and lower relative humidity from open sun to zone three. Compared to the solar tunnel dryer zones, the highest incidence was observed on the coffee drier in the open sun for all the three layer thicknesses.

In the present study, coffee beans dried under different drying zones with different drying layer thickness showed some contamination at the beginning, but with the variation of drying conditions between solar and open sun as well as among different zones of solar drier, variation in terms of incidence was observed for the three identified fungus genera. Initial load of cross-contamination might be the same, but the drying medium condition determines the level of incidence which was low in solar drier than the open sun. [35] reported the occurrence and diversity of these groups of fungi (*Aspergillus*, *Penicillium*, and *Fusarium)* on the surface of coffee cherries and beans as natural coffee contaminants from the field to the warehouse conditions.

**Figure 5:**
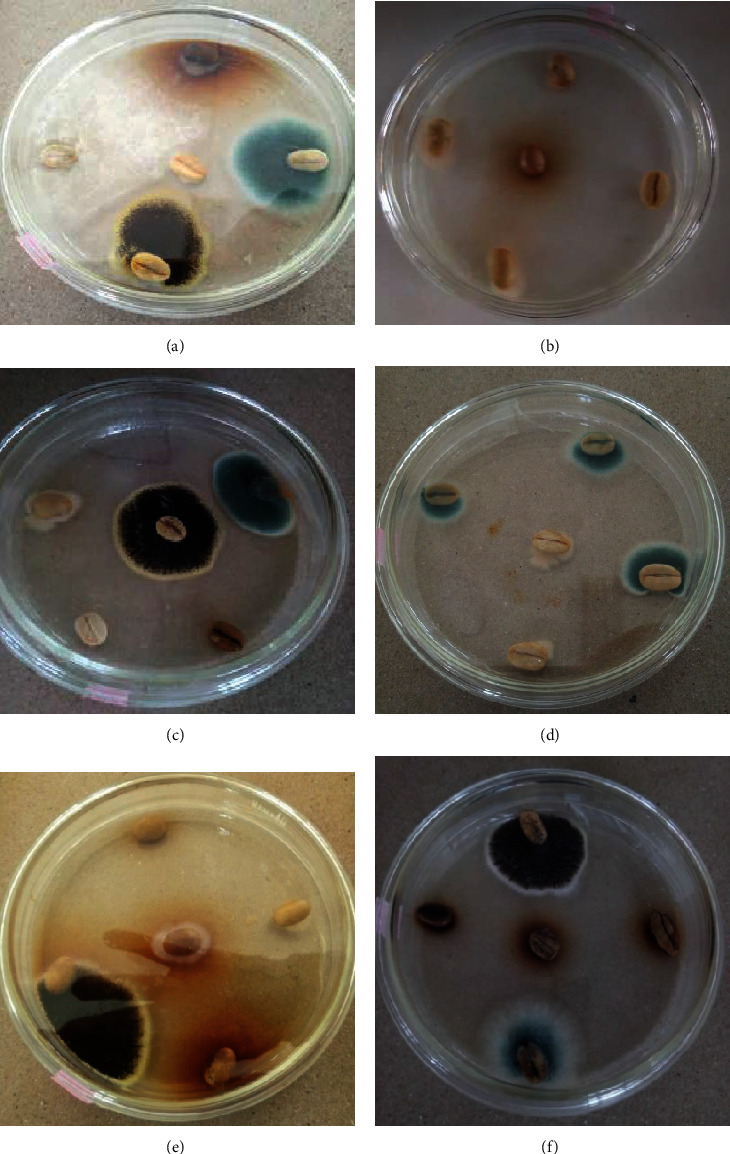
Fungi developed on parchment coffee after 7 days of incubation on PDA at 25°C: (a) open sun, 4 cm; (b) zone 3.2 cm; (c) zone 2.6 cm; (d) zone 1.4 cm; (e) zone 3.6 cm; (f) zone 1.6 cm.

**Figure 6:**
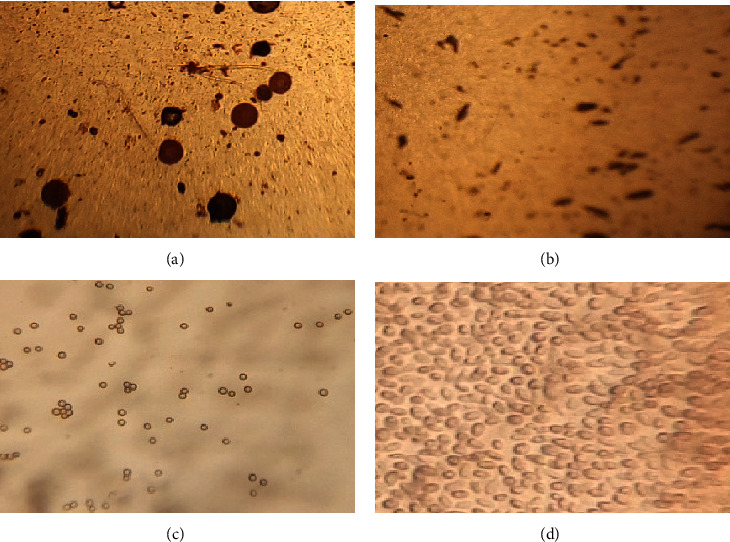
Microscopic view of *Aspergillus* (a), *Fusarium* (b), and *Penicillium* (c, d) genera.

In the current study, minimizing the drying layer thickness reduces the incidence of fungal contamination besides increasing drying temperature (Figures 5 and 6). In line with the present study, Enyan [57] reported that the fungal load increased with increasing depth of bean kernels and ranged from 57% to about 75% while studying the effect of drying method and depth of Robusta coffee.

## 4. Conclusion

Effects of layer thickness and solar tunnel drier zones on drying kinetics on quality and safety of wet-processed parchment coffee (Coffea arabica L.) had been investigated in this study, and the following conclusions can be drawn:

1. Solar tunnel drying is a recommended option for coffee drying by preventing the losses of most of quality parameters by reducing the drying time as compared to commonly used open sun drying method
2. Variations in terms of drying time and moisture content along the long tunnel solar drier can be managed, and a more similar drying time and moisture content of beans can be achieved by applying different layer thicknesses at different zones
3. Among the dryer zones, drier zone two is likely the best drying zone for best coffee quality, but comparative quality grade can be achieved by adjusting drying layer thicknesses at different drier zones

## Figures and Tables

**Table 1:** Drying kinetic models used to determine drying characteristics of parchment coffee in a solar tunnel drier and open sun condition.

No	Model name	Equation	Reference
1	Modified Midilli	MR = exp(−*kt*^*c*) + *bt*	Ghazanfari et al. [17]
2	Diffusion approach	MR = *a*^∗^exp(−*bt*) + (1 − *a*)exp(−*bkt*)	Muhidong and Rahman [18]
3	Modified page	MR = exp(−(*a*.*t*)*b*)	Kingsly et al. [19]
4	Newton	MR = exp(−*a*.*t*)	Muhidong et al. [20]
5	Henderson and Pabis	MR = *a*.exp(−*b*.*t*)	Ibrahim et al. [21]
6	Two terms	MR = *a*.exp(−*b*.*t*) + *k*.exp(−*d*.*t*)	Meisami-Asl et al. [22]
7	Verma	MR = *a*.exp(−*b*.*t*) + (1 − *a*).exp(−*k*.*t*)	Hii et al. [23]

*t* represents elapsed drying time in hours; *a*, *b*, *k*, and *e* are drying constants.

**Table 2:** Influence of drier zones and layer thicknesses on the drying hours of parchment coffee.

Dryer zones	Layer thickness (cm)	Hours of drying	Percent decrease in drying time for specific layer thickness as compared to open sun
Open sun	2	32.7 ± 0.33^d^	0
	4	36.7 ± 0.33^c^	0
	6	47.0 ± 0.00^a^	0

Zone 1	2	25.3 ± 0.33^f^	22.6
	4	28.3 ± 0.33^e^	22.9
	6	39.3 ± 0.33^b^	16.4

Zone 2	2	20.7 ± 0.33^g^	36.7
	4	21.0 ± 0.00^g^	42.8
	6	32.0 ± 0.00^d^	31.9

Zone 3	2	14.0 ± 0.00^i^	57.2
	4	17.3 ± 0.33^h^	52.9
	6	28.3 ± 0.33^e^	39.8

CV%		1.35	—

Values expressed are mean values of three replicates ± standard error. All mean scores, bearing different superscript letters in columns, differ significantly (*p* ≤ 0.05).

**Table 3:** Values of RMSE, *χ*^2^, and *R*^2^ at different layer thicknesses and zones of drier to evaluate the models.

Layer thickness	Zone 1				Zone 2			Zone 3			Open sun drying		
	Models	RMSE	*χ* ^2^	*R* ^2^	RMSE	*χ* ^2^	*R* ^2^	RMSE	*χ* ^2^	*R* ^2^	RMSE	*χ* ^2^	*R* ^2^
2 cm	M. Midilli	0.03026	0.000995	0.9800	0.03896	0.00169	0.9731	0.02682	0.00084	0.9834	0.04335	0.00020	0.9886
	Diffusion approach	0.01796	0.000350	0.9900	0.02465	0.00068	0.9806	0.03221	0.00121	0.9700	0.02662	0.00075	0.9795
	Verma	0.01755	0.000335	0.9887	0.02204	0.00054	0.98522	0.04666	0.00254	0.9537	0.02086	0.00046	0.9848
	Henderson and Pabis	0.01816	0.000359	0.9879	0.02465	0.00068	0.98068	0.04203	0.00206	0.9498	0.02529	0.00068	0.9778
	Newton	0.01879	0.000368	0.9881	0.02466	0.00064	0.98055	0.18988	0.00277	0.9576	0.02662	0.00073	0.9795
	Modified page	0.01879	0.000384	0.9881	0.02600	0.00068	0.98055	0.05075	0.00300	0.9576	0.02662	0.00075	0.9795
	Two terms	0.01816	0.000359	0.9879	0.01908	0.00043	0.98811	0.04203	0.00225	0.9498	0.02529	0.00070	0.9778

4 cm	M. Midilli	0.028610	0.000882	0.9812	0.02252	0.00056	0.98398	0.02080	0.00049	0.9880	0.01682	0.00031	0.9925
	Diffusion approach	0.021354	0.000491	0.9839	0.02844	0.00089	0.97477	0.02199	0.00055	0.9859	0.01457	0.00023	0.9925
	Verma	0.019296	0.000401	0.9868	0.03248	0.00117	0.97847	0.03542	0.00142	0.9795	0.02120	0.00048	0.9861
	Henderson and Pabis	0.021592	0.000502	0.9836	0.03428	0.00118	0.96722	0.03136	0.00111	0.9717	0.02728	0.00079	0.9739
	Newton	0.022166	0.000510	0.9841	0.03928	0.00162	0.97167	0.04109	0.00179	0.9756	0.02975	0.00091	0.9767
	Modified page	0.022166	0.000529	0.9841	0.03928	0.00171	0.97167	0.04109	0.00191	0.9756	0.02217	0.00094	0.9767
	Two terms	0.022773	0.000581	0.9818	0.03261	0.00124	0.96722	0.03859	0.00181	0.9748	0.02728	0.00081	0.9739

6 cm	M. Midilli	0.025642	0.000693	0.9912	0.000624	0.00187	0.97779	0.01973	0.00042	0.9873	0.02249	0.00100	0.9926
	Diffusion approach	0.084907	0.007599	0.8634	0.06017	0.00386	0.97509	0.02479	0.00066	0.9801	0.01012	0.00012	0.9963
	Verma	0.081543	0.007009	0.9702	0.03896	0.00169	0.97309	0.02660	0.00076	0.9845	0.02380	0.00060	0.9873
	Henderson and Pabis	0.029649	0.000927	0.9840	0.024653	0.000675	0.98064	0.02763	0.00082	0.9755	0.02697	0.00076	0.9748
	Newton	0.028976	0.000862	0.9848	0.022036	0.000540	0.98522	0.03377	0.00118	0.9788	0.03185	0.00104	0.9789
	Modified page	0.028976	0.000885	0.9848	0.024649	0.000675	0.98068	0.03377	0.00123	0.9788	0.03185	0.00106	0.9789
	Two terms	0.023345	0.000590	0.98242	0.024661	0.000640	0.98055	0.02763	0.00086	0.9755	0.02697	0.00078	0.9748

**Table 4:** Effective diffusivity of the parchment coffee dried under different drying zones with different thicknesses.

Drier zones	Layer thickness	Diffusivity (m^2^/s)
Open sun	2 cm	1.57*E*-06
	4 cm	1.68*E*-06
	6 cm	1.2*E*-06

Zone 1	2 cm	2.8*E*-06
	4 cm	2.51*E*-06
	6 cm	2.15*E*-06

Zone 2	2 cm	3.48*E*-06
	4 cm	4.12*E*-06
	6 cm	1.98*E*-06

Zone 3	2 cm	5.03*E*-06
	4 cm	2.44*E*-06
	6 cm	1.89*E*-06

**Table 5:** Mean values for the physicochemical property of parchment coffee dried under different drying zones with different layer thicknesses.

Drier zones	Layer thickness (cm)	PH (unit)	TSS (°Brix)	Crude fat (% d.b)	Total polyphenols (GAE/g)	DPPH scavenging capacity (%)	IC_50_ (mg/mL)
Open sun	2	5.6 ± 0.03^cde^	2.7 ± 0.02^cde^	14.9 ± 0.05^a^	46.4 ± 0.02^e^	47.5 ± 0.03^d^	3.3 ± 0.008^g^
	4	5.4 ± 0.02^f^	2.6 ± 0.00^de^	15.2 ± 0.03^a^	48.0 ± 0.02^d^	49.6 ± 0.02^c^	2.7 ± 0.035^h^
	6	5.3 ± 0.01^g^	2.3 ± 0.02^f^	14.7 ± 0.02^ab^	45.9 ± 0.00^f^	47.3 ± 0.01^d^	3.3 ± 0.008^g^

Zone 1	2	5.6 ± 0.05^cd^	2.7 ± 0.01^de^	14.1 ± 0.23^bc^	44.5 ± 0.10^g^	44.3 ± 0.03^f^	4.7 ± 0.144^e^
	4	5.6 ± 0.02^bc^	2.7 ± 0.06^de^	13.7 ± 0.10^cd^	49.4 ± 0.02^c^	50.2 ± 0.02^c^	2.1 ± 0.010^i^
	6	5.7 ± 0.01^b^	2.8 ± 0.12^bcd^	13.5 ± 0.06^cd^	41.1 ± 0.06^i^	40.5 ± 0.05^h^	5.8 ± 0.022^c^

Zone 2	2	5.9 ± 0.01^a^	2.9 ± 0.02^b^	13.7 ± 0.22^cd^	45.9 ± 0.04^f^	45.3 ± 0.04^e^	4.2 ± 0.009^f^
	4	5.6 ± 0.02^cde^	4.3 ± 0.11^a^	13.9 ± 0.12^cd^	53.5 ± 0.05^a^	59.2 ± 0.04^a^	0.8 ± 0.010^k^
	6	5.5 ± 0.02^ef^	2.6 ± 0.01^e^	13.8 ± 0.07^cd^	40.0 ± 0.01^j^	39.7 ± 0.01^h^	6.1 ± 0.042^b^

Zone 3	2	5.6 ± 0.02^de^	2.9 ± 0.02^b^	10.9 ± 0.08^e^	42.5 ± 0.01^h^	41.7 ± 0.02^g^	5.2 ± 0.012^d^
	4	5.6 ± 0.01^cde^	2.8 ± 0.05^bcde^	13.2 ± 0.10^d^	50.6 ± 0.01^b^	55.7 ± 0.01^b^	1.6 ± 0.038^j^
	6	5.6 ± 0.01^bcd^	2.9 ± 0.05^bc^	13.2 ± 0.07^d^	39.2 ± 0.05^k^	38.5 ± 0.04^i^	6.3 ± 0.072^a^

CV%		0.58	2.46	1.16	1.41	1.63	1.54

Values expressed are mean values of three replicates ± standard error. All mean scores, bearing different superscript letters in columns, differ significantly (*p* ≤ 0.05).

**Table 6:** Mean values for sensory property of parchment coffee dried under different drier zones at different layer thickness.

Drier zones	Layer thickness (cm)	Total raw (40%)	Total cup (60%)	Total quality (100%)
Open sun	2	36.3 ± 0.33^a^	48.0 ± 0.0^b^	84.3 ± 0.33^a^
	4	36.7 ± 0.33^a^	48.0 ± 0.0^b^	84.7 ± 0.33^a^
	6	36.7 ± 0.33^a^	48.0 ± 0.0^b^	84.7 ± 0.33^a^

Zone 1	2	36.7 ± 0.33^a^	49.0 ± 1.0^ab^	85.7 ± 1.33^a^
	4	37.0 ± 0.58^a^	49.0 ± 0.0^b^	86.0 ± 0.58^a^
	6	36.3 ± 0.33^a^	48.0 ± 1.0^b^	84.3 ± 0.88^a^

Zone 2	2	36.3 ± 0.33^a^	51.0 ± 0.0^a^	87.3 ± 0.33^a^
	4	36.3 ± 0.33^a^	51.0 ± 0.0^a^	87.3 ± 0.33^a^
	6	36.7 ± 0.33^a^	50.0 ± 1.0^ab^	86.7 ± 1.45^a^

Zone 3	2	36.7 ± 0.67^a^	48.0 ± 0.0^b^	84.7 ± 0.67^a^
	4	37.0 ± 0.33^a^	49.0 ± 0.0^b^	86 ± 0.58^a^
	6	37.0 ± 0.58^a^	48.0 ± 1.0^b^	85 ± 1.15^a^

CV%		1.90	1.18	1.40

Values expressed are mean values of three replicates ± standard error. All mean scores, bearing different superscript letters in columns, differ significantly (*p* ≤ 0.05).

**Table 7:** Influence of drying zones and layer thickness on fungal incidence and infection of the parchment coffee.

Drying zones	Layer thickness (cm)	Fungal count			Mean fungal infection %
		Aspergillus	Penicillium	Fusarium	
Sun	2	17	3	2	45.3 ± 1.33^c^
	4	33	4	3	77.3 ± 2.67^ab^
	6	30	12	5	93.3 ± 1.33^a^
	Sum	80	19	10	

Zone 1	2	7	2	1	14.7 ± 1.76^efg^
	4	12	5	2	38.7 ± 2.00^cd^
	6	28	8	5	82.7 ± 1.33^ab^
	Sum	47	15	8	

Zone 2	2	2	1	0	5.3 ± 1.33^fg^
	4	10	3	0	28.0 ± 1.15^cde^
	6	32	0	5	73.3 ± 1.33^b^
	Sum	44	4	5	

Zone 3	2	2	0	0	4.0 ± 1.15^g^
	4	1	1	2	6.7 ± 0.67^fg^
	6	12	2	0	23.3 ± 0.67^def^
	Sum	15	3	2	
	Total sum	186	41	25	

CV%					9.98

Values expressed are mean values of three replicates ± standard error. All mean scores, bearing different superscript letters in columns, differ significantly (*p* ≤ 0.05).

## Data Availability

The datasets supporting the conclusions of this article are included within the article and could be available from the corresponding authors upon request.
